# Role of IL-38 and Its Related Cytokines in Inflammation

**DOI:** 10.1155/2015/807976

**Published:** 2015-03-19

**Authors:** Xianli Yuan, Xiao Peng, Yan Li, Mingcai Li

**Affiliations:** Zhejiang Provincial Key Laboratory of Pathophysiology, Department of Immunology, Ningbo University School of Medicine, Ningbo 315211, China

## Abstract

Interleukin- (IL-) 38 is a recently discovered cytokine and is the tenth member of the IL-1 cytokine family. IL-38 shares structural features with IL-1 receptor antagonist (IL-1Ra) and IL-36Ra. IL-36R is the specific receptor of IL-38, a partial receptor antagonist of IL-36. IL-38 inhibits the production of T-cell cytokines IL-17 and IL-22. IL-38 also inhibits the production of IL-8 induced by IL-36*γ*, thus inhibiting inflammatory responses. IL-38-related cytokines, including IL-1Ra and IL-36Ra, are involved in the regulation of inflammation and immune responses. The study of IL-38 and IL-38-related cytokines might provide new insights for developing anti-inflammatory treatments in the near future.

## 1. Introduction

Our understanding of the interleukin-1 family (IL-1F) has recently expanded to encompass 11 members: IL-1F1-IL-1F11 [1]. These cytokines are also termed IL-1*α*, IL-1*β*, IL-1 receptor antagonist (IL-1Ra), IL-18, IL-36Ra, IL-36*α*, IL-37, IL-36*β*, IL-36*γ*, IL-38, and IL-33, respectively [2] (Table 1). These protein molecules play a prominent role in inflammation and immune responses, acting as the first line of defense against invasive pathogenic microorganisms and physical damage. IL-38 is a novel member of the IL-1F identified in 2001 by a unique high throughput cDNA screening approach taking advantage of a set of oligonucleotide probes to hybridize successive arrays of human cDNAs from various tissues [3, 4]. The former name of IL-38 is IL-1HY2, similar to IL-36Ra (IL-1HY1). IL-38 is the 10th member of IL-1F and its receptor is termed IL-1 receptor-related protein 2 (IL-1Rrp2, IL-36R). IL-38 is an IL-36 antagonist and functions as a typical receptor antagonist similar to IL-1Ra and IL-36Ra [5]. IL-38 reduces inflammation by preventing the binding of agonist receptor ligands to IL-36R, a specific receptor of IL-38.

## 2. Biological Characteristics of IL-38

The novel IL-1-like gene,* IL-38*, is located in the IL-1 family cluster (except* IL-18* and* IL-33*) on human chromosome 2q13-14.1 near the* IL-1Ra* gene (*IL-1RN*) and* IL-36Ra* gene (*IL-36RN*) [6]. The* IL-38* gene is located 49,479 bp upstream from* IL-1RN* on the same DNA strand [7].* IL-38* shares high sequence homology with* IL-1Ra* and* IL-36Ra*. The primary translated product is an IL-38 precursor, 152 amino acids in length and with 16.9 kD molecular mass. Sequence analysis indicated that the IL-38 protein shares 41% homology with IL-1Ra and 43% homology with IL-36Ra [4, 7] and lower homology (14–30%) with IL-1*β* and other IL-1 family proteins. In mammalian Chinese hamster ovary cells, recombinant IL-38 protein was synthesized into two forms, a major form at 25 kD and a minor form at 17 kD. Lin et al. [3] suggested that the major form of IL-38 might be a result of posttranslational protein modifications, such as phosphorylation. However, studies have shown that the IL-38 protein lacks N-glycosylation and O-glycosylation consensus sites in Chinese hamster ovary cells [3]. As is typical of the IL-1 family, including IL-36Ra, IL-36*α*, IL-36*β*, and IL-36*γ*, IL-38 lacks a signal peptide and caspase-1 consensus cleavage site [3, 7]. Furthermore, the natural N terminus for IL-38 is still unclear [7]. Using the multiple alignment sequence profile-based searching method (PSI-BLAST), an automated sequence and structure searching procedure (high throughput modeling), and a fold recognition method (SeqFold), three-dimensional structural models of IL-38 were predicted. The IL-38 structural model displays a 12-*β*-stranded trefoil structure and shares similarity with the crystal structure of IL-1Ra and IL-1*β* [3, 8]. The characteristics of IL-38 provide further evidence that it belongs to the IL-1F. In addition, IL-38 was categorized in the IL-36 subfamily according to the length of its precursor. Thus, IL-38 likely also belongs to this subfamily because it has the ability to bind to the common receptor IL-36R [2, 9].

**Figure 1:**
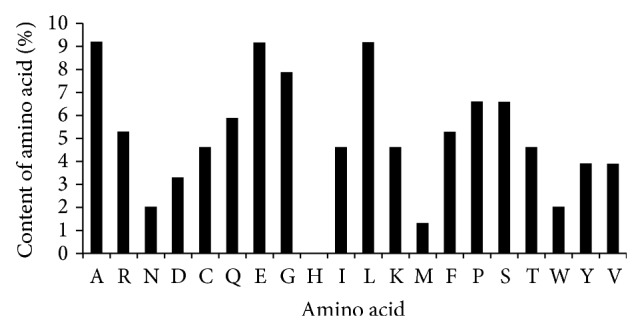
Amino acid composition of human IL-38 protein.

To date, no reports have described the components and properties of IL-38. We adopted the ProtParam tool [14, 15] application to analyze the amino acid composition of IL-38, which consists of 19 amino acids. A (alanine), E (glutamic acid), and L (leucine) were the most prevalent amino acids (9.2%) in IL-38, followed by G (glycine, 7.9%), P (proline, 6.6%), and S (serine, 6.6%). Only H (histidine) was not present in IL-38 (Figure 1). The molecular weight of IL-38 is 16.9 kD and is consistent with a previous study, as predicted by ProtParam tool. IL-38 has a half-life of 7 h, an isoelectric point (pI) of 4.94, and the molecular formula of C_757_H_1164_N_198_O_226_S_9_, as analyzed by SOPMA (self-optimized prediction method from alignment) [15, 16]. Moreover, the second structure of IL-38 was composed of a *β*-turn, random coil, *β*-sheet, and *α*-helix. Analysis further indicated that the random coil and *β*-sheet were uniformly distributed in the protein chain.

By multitissue first-strand cDNA PCR analysis, IL-38 mRNA was measured in a range of tissues, including heart, placenta, fetal liver, skin, spleen, thymus, and tonsil. IL-38 was expressed mostly in the skin and in proliferating B cells of the tonsil [3]. However, in nonimmune tissues, such as human heart and placenta, IL-38 was present at low levels, similar to other IL-1F members [7]. Some IL-1F members are constitutively produced, whereas others have inducible expression, being rapidly induced by bacteria or inflammatory mediators [7, 17, 18]. The expression type of IL-38 is currently unknown and requires further research.

## 3. Receptor and Signaling Pathway of IL-38

In 2001, it was speculated that IL-38 acted as an IL-1 receptor antagonist because of its amino acid homology to the naturally occurring IL-1Ra and the observation that IL-38 could bind to the soluble IL-1 receptor type I (IL-1RI). IL-1RI was once considered a receptor for IL-38 [3, 5]. However, the binding affinity of recombinant IL-38 is significantly lower than that of IL-1Ra and IL-1*β*. Recently, researchers doubted whether IL-1RI was a receptor for IL-38. IL-1Rrp2 was regarded as an IL-38-specific receptor and was also called IL-36R. In a report by van de Veerdonk [5], the combining capacity between IL-38 and IL-1RI, IL-36R, IL-18R, and IL-1R accessory proteins (IL-1RAP, IL-1RAcP) was compared, in the presence of increasing concentrations of IL-38. IL-38 bound to IL-36R but did not bind to the other immobilized receptors. Furthermore, IL-38 binding to immobilized IL-36R was comparable to IL-36Ra binding to the same receptor. It was observed that increasing the concentration of IL-38 resulted in increased optical density, reaching a plateau at 16.7 *μ*g/mL, a higher value than that obtained for IL-36Ra. Based on the binding studies, these data suggest IL-38 could act by blocking the IL-36R pathway.

**Figure 2:**
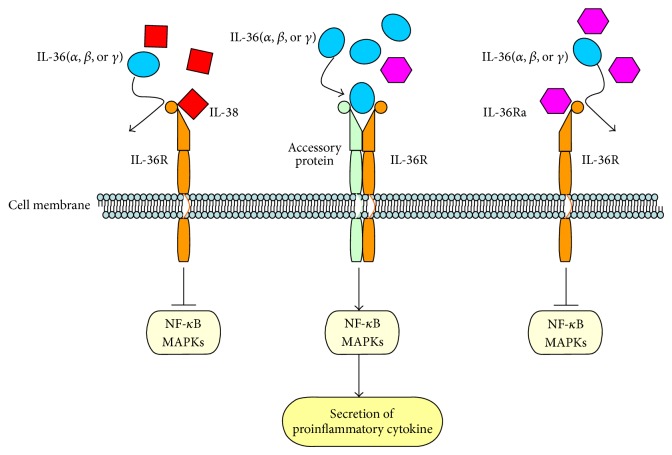
Receptor and signaling pathway of IL-38.

The most recently identified IL-1 family members are widely expressed in inflammatory cells. These cytokines combine with the cell-surface receptor IL-1R and induce downstream signaling, including downstream nuclear transcripts such as nuclear factor-*κ*B (NF-*κ*B) and activator protein-1 (AP-1). Furthermore, as a feedback and adjustment mechanism, these signaling molecules induced the expression of cyclooxygenase, nitric oxide synthase, and other inflammatory mediators to promote the development of inflammation [19, 20]. In line with the characteristics of IL-38 and the homology of IL-38 and IL-36Ra, it can be concluded that IL-38 has a role in inflammatory disease by IL-36Ra pathway-related molecules (Figure 2). The biological function of IL-38 is to inhibit IL-36 cytokine (IL-36*α*, IL-36*β*, and IL-36*γ*) binding to IL-36R, similar to IL-36Ra. According to its activity as a receptor antagonist, IL-38 may have an anti-inflammatory function. IL-38 might also be related to IL-1R and IL-18R signaling pathways, although there is no evidence regarding its role in these specific signaling pathways.

## 4. Biological Activity of IL-38 and Related Cytokines

Because of its homology with other IL-1F members, IL-38 is thought to have the biological activity of IL-1F members. IL-1 cytokines are primarily proinflammatory cytokines as they stimulate the expression of genes associated with inflammation and immunological diseases. IL-1*α* or IL-1*β* binds to its primary receptor IL-1RI, which recruits a second receptor subunit, IL-1RAcP. Formation of the receptor heterodimer induces biological responses typically involving the activation of NF-*κ*B and mitogen-activated protein kinase (MAPK) pathways [21]. IL-1F6 (IL-36*α*), IL-1F8 (IL-36*β*), and IL-1F9 (IL-36*γ*) also activate NF-*κ*B and MAPKs similar to IL-1. Therefore, most molecules involved in IL-1F-induced signaling, such as cytokines, chemokines, adhesion molecules, and enzymes, are mediators of inflammatory diseases [22, 23].

### 4.1. IL-1Ra

IL-1Ra (IL-1F3) is the receptor antagonist of IL-1, a protein composed of two major subunits, IL-1*α* and IL-1*β* [24]. IL-1Ra is synthesized and released in response to the same stimuli that lead to IL-1 production. IL-1Ra, a potent anti-inflammatory cytokine, competitively inhibits stimulation by inflammatory mediators by binding to IL-1R1 and preventing the recruitment of IL-1RAcP. IL-1Ra is associated with severe autoimmune and inflammatory diseases such as periodontitis [25], vaginitis [26], non-Hodgkin's lymphoma [27], gastric cancer [28, 29], osteoarthritis [30], precancerous lesions [31], and inflammatory bowel diseases [32]. Deficiency of the IL-1-receptor antagonist (DIRA), caused by mutations in* IL1RN*, can lead to an autosomal recessive autoinflammatory disease. DIRA allows unopposed actions of IL-1, resulting in life-threatening excessive systemic IL-1-mediated inflammation with skin and bone involvement [33]. A study by Korthagen et al. aimed to elucidate the influence of polymorphisms in* IL1RN* on idiopathic pulmonary fibrosis (IPF) susceptibility and mRNA expression. Polymorphisms of* IL1RN* manifested as a variable number tandem repeat (VNTR), which affected IL-1Ra mRNA expression, suggest that lower levels of IL-1Ra predispose to developing IPF [34]. In addition,* IL-1RA* VNTR may be associated with Parkinson's disease risk [35].* IL1RN* may be a future novel therapeutic target with high specificity, low toxicity, and side effects for the treatment of specific diseases. It is important to enhance our understanding of* IL1RN* functions, such as its interaction with other genes and the influence of environmental factors on its production, to develop treatments with reduced side effects [34].

In addition, IL1Ra-deficient (IL-1Ra−/−) mice, good animal models for experimental studies, spontaneously develop several inflammatory diseases, resembling arthritis [36], aortitis [37], intervertebral disc degeneration [38], and psoriasis in humans due to unopposed excess IL-1 signaling. The current knowledge also suggested that IL-1Ra, an endogenous inhibitor of IL-1, related to alcoholic steatohepatitis [39], liver damage [40], lung damage [41], fat mass [42], and aqueous-deficient dry eye in autoimmune diseases [43].

### 4.2. IL-36Ra

IL-36Ra (IL-1F5) shares 44% homology with IL-1Ra and is an antagonist of  IL-36*α*, IL-36*β*, and IL-36*γ*. IL-36Ra has a *β*-stranded trefoil structure, similar to other family members of IL-1, with a conserved hydrophobic core. IL-36Ra binds to IL-1Rrp2 and has biological effects on immune cells. The mechanism of IL-36Ra antagonism is analogous to IL-1Ra by forming a functional signaling complex. IL-36Ra protein starting at Val-2 is fully active and inhibits IL-36*α*, IL-36*β*, and IL-36*γ*. The A-X-Asp motif is conserved in all IL-1F members at the N-terminal, where Val-2 of IL-36Ra lies in this 9-amino acid conserved sequence. All four IL-36 cytokines lack a conventional signal sequence and function as extracellular cytokines although it is unclear how they are secreted [44]. In addition, IL-36Ra antagonist activity requires removal of the N-terminal methionine present in the primary translation product [45]. More extensive N-terminal amino truncation of  IL-36*α*, IL-36*β*, and IL-36*γ* can dramatically increase their specific activities [45].

IL-36Ra plays a key role in innate and adaptive immunity by stimulating helper T-cell responses and it is associated with many inflammatory diseases. Recessive homozygous mutations in* IL36RN* are the major cause for the development of generalized pustular psoriasis [46–49]. Mutations in* IL36RN*, including premature termination codon mutations, frameshift mutations, and substitutions of amino acid lead to incorrect folding of the IL-36Ra protein. These mutations inhibit the activity of IL-36Ra, thus failing to antagonize IL-36 signaling pathways and inducing inflammatory skin disease due to the high levels of IL-36*α*, IL-36*β*, and IL-36*γ*. Excessive IL-36 levels in the skin of mice lead to symptoms to human psoriasis. However, IL-36Ra-deficient mice develop the more serious pustular psoriasis. Therefore, treatment with a combination of IL-36Ra and IL-36R might improve psoriasis by inhibiting IL-36 stimulation and might be an ideal treatment strategy for inflammation of human skin. IL-36Ra functions as an anti-inflammatory cytokine in the brain [50] and enhances the hippocampal expression of IL-4. This is a consequence of its interaction with the orphan receptor, single Ig IL-1R-related molecule (SIGIRR)/TIR8. Collectively,* in vitro* IL-4 mRNA and protein expression in glia induced by the interaction of IL-36Ra and SIGIRR/TIR8 play a critical role in its anti-inflammatory properties [5]. IL-36 cytokines also have a significant association in the pathogenesis of rheumatoid arthritis [9, 51, 52], inflammatory lung diseases [53, 54], obesity [55], bile duct occlusion disorder, and chronic glomerulonephritis [56]. Data strongly suggest that IL-36Ra might be a useful treatment for IL-36-related diseases.

### 4.3. IL-38

In recent years, scholars identified a novel CD4^+^ T-cell subtype, which was different from T-helper 1 (Th1) and Th2 cells. These cells were named Th17 cells due to the expression of IL-17 and these discoveries have improved our understanding of inflammatory processes. Th17 cells are different from natural T-cell precursors, and the mature cells secrete a variety of cytokines such as IL-17 and IL-22 [57, 58]. Th17 plays an important role in a variety of autoimmune diseases and has an independent regulatory mechanism for their differentiation and development. Th17 is associated with the pathogenesis of systemic lupus erythematosus, rheumatoid arthritis, multiple sclerosis, psoriasis, inflammatory bowel disease, and autoimmune thyroid diseases [44]. Previous studies demonstrated the functions of IL-38 and Th17 cells by blocking the IL-1R, IL-18R, and IL-36R pathways [5]. These data suggested that the influence of IL-38 on Th17 cells was similar to blocking IL-1R and IL-36R pathways, which suppressed IL-17 and IL-22 secretion. Consistent with binding data and the suppression of IL-17 and IL-22, we suggest that IL-38 has similar biological effects on Th17 cells.

*IL-38* gene polymorphisms are associated with psoriatic arthritis (PsA), ankylosing spondylitis (AS) [59–61], and cardiovascular disease [62], suggesting that IL-38 is strongly correlated with these inflammatory diseases. The frequencies of Th17 cells are significantly increased in the peripheral blood of patients with PsA and AS [8, 63–68]. In addition, the number of Th17 cells and serum IL-17 levels were strongly related to systemic disease activity both at the onset and during disease progression of  PsA and AS [68]. IL-38 reduced the expression of* C. albicans*-induced IL-17 and IL-22 from peripheral blood mononuclear cells (PBMCs) by reducing the stimulation of proinflammatory cytokines in the tissues. A recent study reported that low concentrations of IL-38 were more effective than higher concentrations in inhibiting IL-17 and IL-22 production because higher concentrations modestly increased IL-22 [5].

Similar to IL-36Ra, blocking IL-38 suppressed* C. albicans*-induced Th17 cytokine production [5, 69]. Both IL-38 and IL-36Ra inhibited the production of IL-17 and IL-22 by specifically binding to the cell surface-specific protein receptor IL-36R. However, neither IL-38 nor IL-36R functions as a classic receptor antagonist. In PBMCs, the dose-response suppression of IL-38 and IL-36Ra by IL-36*γ*-derived IL-8 was not similar to that of IL-1Ra. Neutrophils and T cells in inflammatory tissues are attracted by IL-8, a chemokine. IL-38 decreased the production of proinflammatory cytokines similar to IL-36Ra [5, 70]. In contrast, IL-38 and IL-36Ra have parallel effects on the production of lipopolysaccharide-induced IL-6 from dendritic cells (DCs), inducing a twofold increase [5]. IL-6 has two adverse effects on immune cells: IL-6 is proinflammatory but also suppresses inflammation in tissues injured by burns or other damages.

IL-1Ra and IL-38 have a comparable dose-effect regarding their antagonist activities and function as classic receptor antagonists; the higher the concentration of IL-22, the stronger its inhibition. Compared with IL-1Ra and IL-38, IL-36Ra does not behave as a typical receptor antagonist. IL-38 and IL-36Ra function as antagonists at high concentrations, but at low concentrations, they inhibit the binding of coreceptors. Thus, IL-38 and IL-36Ra are defined as partial receptor antagonists, although they mimic the effects of IL-1Ra on the production of inflammatory cytokines.

## 5. Future Perspectives

IL-1 and most related family members are primarily proinflammatory cytokines that induce the expression of genes associated with inflammatory diseases. Only IL-37 acts as an anti-inflammatory cytokine. The binding of IL-1Ra and IL-36Ra to their receptor reduces inflammation by blocking the binding of receptor ligands. The production of fungal-induced IL-17, IL-22, and IL-36*γ*-derived IL-8 was decreased by IL-38, which may play an important anti-inflammatory role in inflammatory diseases. Many articles have demonstrated that IL-1Ra and IL-36Ra are associated with arthritis and psoriasis, respectively. In addition, IL-38 can specifically bind to IL-36R, similar to IL-36Ra. IL-36 cytokine has significant* in vivo* effects on DCs and T cells in human immune responses via its role in the differentiation of inflammatory Th1 cells [70–72].

In conclusion, the current knowledge supports the concept that IL-38 may be closely associated with IL-36-mediated inflammatory diseases. Thus far, the IL-1 receptor antagonist anakinra, the soluble decoy receptor rilonacept, and the neutralizing monoclonal anti-IL-1*β* antibody canakinumab have been approved as IL-1-targeting agents for the treatment of specific diseases. Another study demonstrated the beneficial use of a monoclonal antibody directed against the IL-1 receptor and a neutralizing anti-IL-1*α* antibody in clinical trials [73]. The IL-38-related signaling pathway is poorly understood and requires further study. Furthermore, the function and mechanism of IL-38-related diseases remain elusive and awaits elucidation. The increasing knowledge of the mechanisms that regulate chronic inflammatory conditions such as rheumatoid arthritis may provide a potential strategy for the development of anti-inflammatory treatments for autoimmune diseases and establish a theoretical basis for clinical trials and drug development.

## Figures and Tables

**Table 1:** IL-1 family members [10, 11].

Cytokine	Family name	Receptor	Coreceptor	Property
IL-1*α*	IL-1F1	IL-1RI	IL-1RAcp	Proinflammatory
IL-1*β*	IL-1F2	IL-1RI	IL-1RAcp	Proinflammatory
IL-1Ra	IL-1F3	IL-1RI	NA	Antagonist for IL-1*α*, IL-1*β*
IL-18	IL-1F4	IL-18R*α*	IL-18R*β*	Proinflammatory
IL-36Ra	IL-1F5	IL-36R	NA	Antagonist for IL-36*α*, IL-36*β*, and IL-36*γ*
IL-36*α*	IL-1F6	IL-36R	IL-1RAcp	Proinflammatory
IL-37	IL-1F7	IL-18R*α*?	Unknown	Anti-inflammatory, transcription regulating factor [12]
IL-36*β*	IL-1F8	IL-36R	IL-1RAcp	Proinflammatory
IL-36*γ*	IL-1F9	IL-36R	IL-1RAcp	Proinflammatory
IL-38	IL-1F10	IL-36R	Unknown	Antagonist for IL-36*α*, IL-36*β*, and IL-36*γ*
IL-33	IL-1F11	ST2	IL-1RAcp	Proinflammatory, transcription regulating factor [13]

NA: not applicable; ?: requires confirmation.
